# Test–retest reliability of motion capture technology for assessing balance in children with autism spectrum disorder

**DOI:** 10.3389/fneur.2026.1821167

**Published:** 2026-07-22

**Authors:** Tingting Ma, Lingyun Jia, Amei Feng, Qi Zhang, Lin Li, Yuxi Qin, Shujin Hao

**Affiliations:** 1 Department of Physical Therapy for Children, China Rehabilitation Research Center, Beijing Boai Hospital, Beijing, China; 2 Rehabilitation Medical College, Capital Medical University, Beijing, China; 3 School of Physical Education, Renmin University of China, Beijing, China; 4 Sichuan Provincial Bayi Rehabilitation Center, Chengdu, Sichuan, China; 5 Beijing Haolantongxu Technology & Trade Co., Ltd., Beijing, China

**Keywords:** autism spectrum disorder, child, motion capture, postural balance, reproducibility of results

## Abstract

**Background:**

Children with autism spectrum disorder (ASD) often show balance impairments that may affect motor development. Portable motion capture may provide quantitative balance indices, but its test–retest reliability in pediatric ASD remains unclear.

**Research question:**

This study evaluated seven-day test–retest reliability of a portable marker-based motion capture system for head-sway-derived balance indices in children with ASD and described exploratory unadjusted differences from typically developing (TD) children.

**Methods:**

Twenty-two children (11 ASD, 11 TD) completed long-sitting, eyes-open standing, and eyes-closed standing balance tests using head-mounted reflective markers. Outcomes were mediolateral sway amplitude (Dx), anteroposterior sway amplitude (Dy), and 95% confidence ellipse area (Area). Reliability was assessed using intraclass correlation coefficients, standard error of measurement, and Bland–Altman plots. Between-group analyses were exploratory because the groups were not matched by age or sex.

**Results:**

In children with ASD, Dy showed good reliability (ICC = 0.77–0.87; SEM = 1.98–3.19), with the highest reliability in long sitting (ICC = 0.87). Dx showed poor to moderate reliability (ICC = 0.45–0.69), and Area showed moderate to good reliability (ICC = 0.65–0.77). Exploratory comparisons showed larger sway values in the ASD group, but these results may reflect baseline age and sex differences.

**Significance:**

The system reproducibly measured anteroposterior head-sway in children with ASD, particularly in long sitting. Further validation against reference balance measures and larger age- and sex-matched studies is needed before diagnostic or discriminative use.

## 1 Introduction

Autism spectrum disorder (ASD) is characterized by deficits in social communication and interaction, together with restricted and repetitive behaviors (1). Beyond these diagnostic features, motor impairments are common in ASD and include deficits in balance, gait, praxis, and coordination (2). Postural control difficulties in ASD have been linked to symptom severity and to kinematic alterations of the trunk, pelvis, and lower limbs that may increase fall risk (3). Balance is a foundation for sitting and standing postural control and supports the acquisition and execution of motor skills (4). Children with ASD often demonstrate weaker balance performance than typically developing (TD) peers, which may restrict gross motor activities and age-appropriate participation (5).

Balance in children with ASD has been assessed using walking tasks, plantar pressure systems, clinical scales, force platforms, and wearable inertial measurement units (IMUs) (6–9). Each approach has advantages and limitations. Clinical scales are practical but may rely on examiner judgment, show restricted measurement resolution, and be susceptible to floor or ceiling effects (7). Force-platform stabilometry provides precise quantitative information but typically requires dedicated laboratory equipment, standardized acquisition conditions, and limited portability (9). Wearable IMU-based assessment is more portable and can support repeated testing, but it remains sensitive to sensor placement, algorithm selection, and population-specific validation (8). These limitations highlight the need for quantitative tools that are sufficiently reliable for pediatric ASD assessment while remaining feasible in clinical environments.

Vision-based and marker-based motion capture systems record the movement of body landmarks and convert those movements into analyzable kinematic data (10). Motion capture has been used to quantify gait, dynamic stability, and locomotor development in TD children (11, 12), and to characterize movement impairments in pediatric neurological conditions such as cerebral palsy (13, 14). Portable systems may reduce some barriers associated with laboratory-based systems by allowing assessment in familiar clinical settings, but their measurement properties must be established before the resulting indices can be used in research or clinical decision-making.

Despite this progress, evidence supporting portable motion capture for balance assessment in children with ASD remains limited. Existing work has more often focused on gait or upper extremity movement than on test–retest reliability of balance indices across clinically relevant postural conditions. In addition, using head-mounted markers provides a pragmatic measure of head-sway behavior rather than a direct measure of whole-body center of mass. Reliability of the captured signal and validity of the underlying construct are therefore distinct questions. Establishing reliability is a necessary first step, while construct and concurrent validity require comparison with reference methods such as force platforms or full-body motion capture.

The present study was designed to evaluate a newly developed portable marker-based motion capture system for pediatric balance assessment. The primary aim was to determine test–retest reliability across a seven-day interval in children with ASD under three conditions: long-sitting, eyes-open standing, and eyes-closed standing. The secondary aim was to describe reliability in TD children using the same protocol. A further exploratory aim was to compare unadjusted balance indices between ASD and TD groups; these comparisons were interpreted cautiously because the study was not powered for group differences and the groups were not matched for age or sex. We hypothesized that anteroposterior sway measures would show at least moderate reliability, with reliability varying by postural condition.

## 2 Methods

### 2.1 Study design and ethics

This prospective test–retest reliability study used a repeated-measurement design with a seven-day interval between sessions. The seven-day interval was selected to minimize immediate practice effects while remaining short enough to assume stable balance ability in the absence of intervention, consistent with reliability protocols for stabilometry and accelerometry-based postural stability assessment (9, 15). The primary focus was reliability estimation; any between-group differences were considered exploratory.

The study protocol was approved by the Medical Ethics Committee of the China Rehabilitation Research Center (Ethics Approval No. 2024–008-2). All procedures complied with the Declaration of Helsinki and relevant institutional guidelines. Before participation, the experimental procedures were explained to all children and their legal guardians, and written informed consent was obtained from each child’s legal guardian.

### 2.2 Participants

Sample size calculation was performed using PASS 15.0 software with statistical power set to 0.80 and alpha level set to 0.05. Based on reliability reporting recommendations, the target ICC value was set at 0.75 (16). The calculated required sample size was 11 participants per group, giving a total target sample of 22 participants. This calculation addressed the primary reliability aim only; the study was not powered to make definitive between-group inferences. Participant recruitment and baseline characteristics are reported in Results section 3.1 and Tables 1–3.

**Table 1:** Basic information of children with ASD.

Number	Gender	Age (years)	Height (cm)	Weight (kg)	BMI (kg/m^2^)
1	Male	6.00	121.0	22.10	15.10
2	Male	6.00	113.5	18.30	14.21
3	Male	4.83	114.0	21.24	16.31
4	Male	6.08	121.5	29.76	20.20
5	Male	3.58	106.5	17.74	15.60
6	Female	4.00	106.5	18.96	16.70
7	Male	3.50	102.5	17.80	16.90
8	Male	6.00	120.0	25.10	17.40
9	Male	3.92	108.0	18.00	15.40
10	MALE	7.42	127.0	27.10	16.80
11	Female	3.00	103.0	17.70	16.70

**Table 2:** Basic information of TD children.

Number	Gender	Age (years)	Height (cm)	Weight (kg)	BMI (kg/m^2^)
1	Male	6.75	126.5	33.50	20.9
2	Female	4.83	105.0	14.20	12.9
3	Male	8.42	129.0	23.70	14.2
4	Male	8.42	142.0	32.25	16.0
5	Female	10.75	154.5	35.80	15.0
6	Female	6.83	129.0	37.40	22.5
7	Female	6.08	114.5	19.80	15.1
8	Male	9.42	135.0	30.95	17.0
9	Male	5.00	113.0	19.80	15.5
10	Female	9.50	142.0	26.00	12.9
11	Female	8.42	118.5	19.25	13.7

**Table 3:** Basic information of children.

Information	TD children	Children with ASD	Test statistic	*p*-value
Age (years)	7.67 ± 1.92	4.94 ± 1.43	*t* = 3.62	0.002**
Height (cm)	128.09 ± 14.74	113.05 ± 8.38	*t* = 2.76	0.012*
Weight (kg)	26.61 ± 7.8	21.26 ± 4.29	*t* = 2.08	0.051
BMI (kg/m^2^)	15.97 ± 3.12	16.48 ± 1.55	*t* = 0.70	0.49
Male (%)	45.45	81.82	χ^2^ = 3.27	0.07
Female (%)	54.55	18.18		

*p < 0.05, **p < 0.01. Age and sex distribution differed between groups and were considered potential confounders in exploratory between-group comparisons.

Inclusion criteria for children with ASD included:

1. Meeting diagnostic criteria for ASD according to the Chinese Classification of Mental Disorders, Third Edition (CCMD-3) (17), with diagnosis confirmed through comprehensive clinical evaluation.
2. Demonstration of postural control impairments as identified through the Evaluation in Ayres Sensory Integration (EASI) assessment (18).
3. Sufficient cognitive ability to comprehend and cooperate with testing instructions.
4. Voluntary participation with written informed consent provided by legal guardians.

Exclusion criteria for children with ASD included:

1. Presence of major medical conditions such as congenital heart disease or organ failure.
2. Comorbid psychiatric disorders beyond ASD.
3. Comorbid neurological conditions including epilepsy or cerebral palsy.
4. Inability to comprehend the majority of testing instructions or difficulty cooperating to complete the assessment protocol.

Inclusion criteria for TD children included:

1. Absence of diagnosed developmental, neurological, or psychiatric conditions.
2. Ability to comprehend and follow testing instructions.
3. Voluntary participation with written informed consent from legal guardians.

Exclusion criteria for TD children included:

1. History of motor impairments or movement disorders.
2. Diagnosed learning disabilities or developmental delays.

TD children were recruited from the local community by convenience sampling. No age- or sex-matching procedure was applied; therefore, baseline comparability was evaluated statistically and between-group analyses were interpreted as exploratory.

### 2.3 Instrumentation

**Figure 1:**
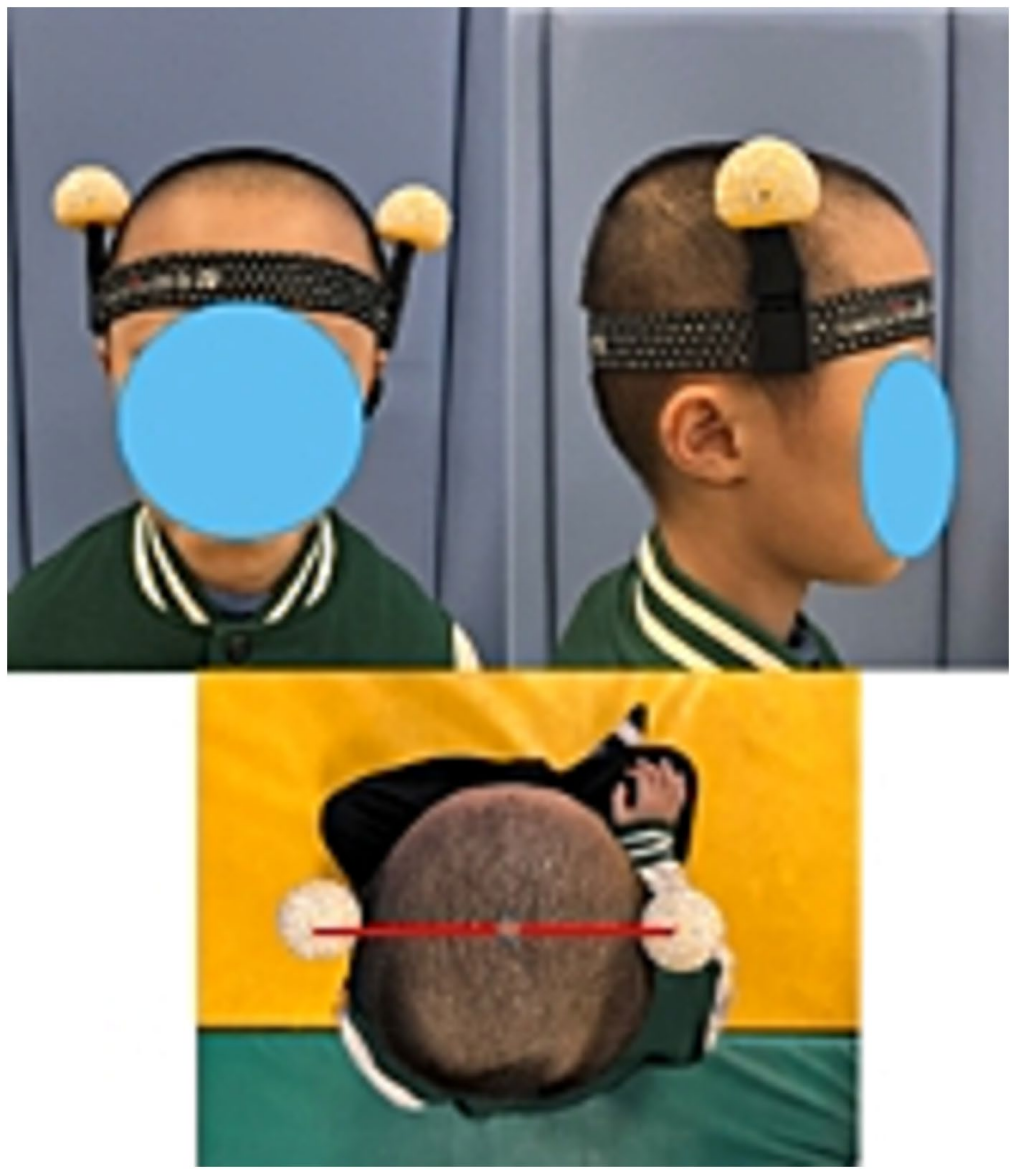
Head marker balls used during testing. **(A)** Anterior view, **(B)** lateral view, and **(C)** superior view.

Participants wore bilateral head-mounted marker balls (Figure 1) to enable tracking of head movements. The midpoint between the head markers was used as a head-motion reference point. This reference point was used to derive head-sway indices and was not assumed to represent the true whole-body center of mass.

**Figure 2:**
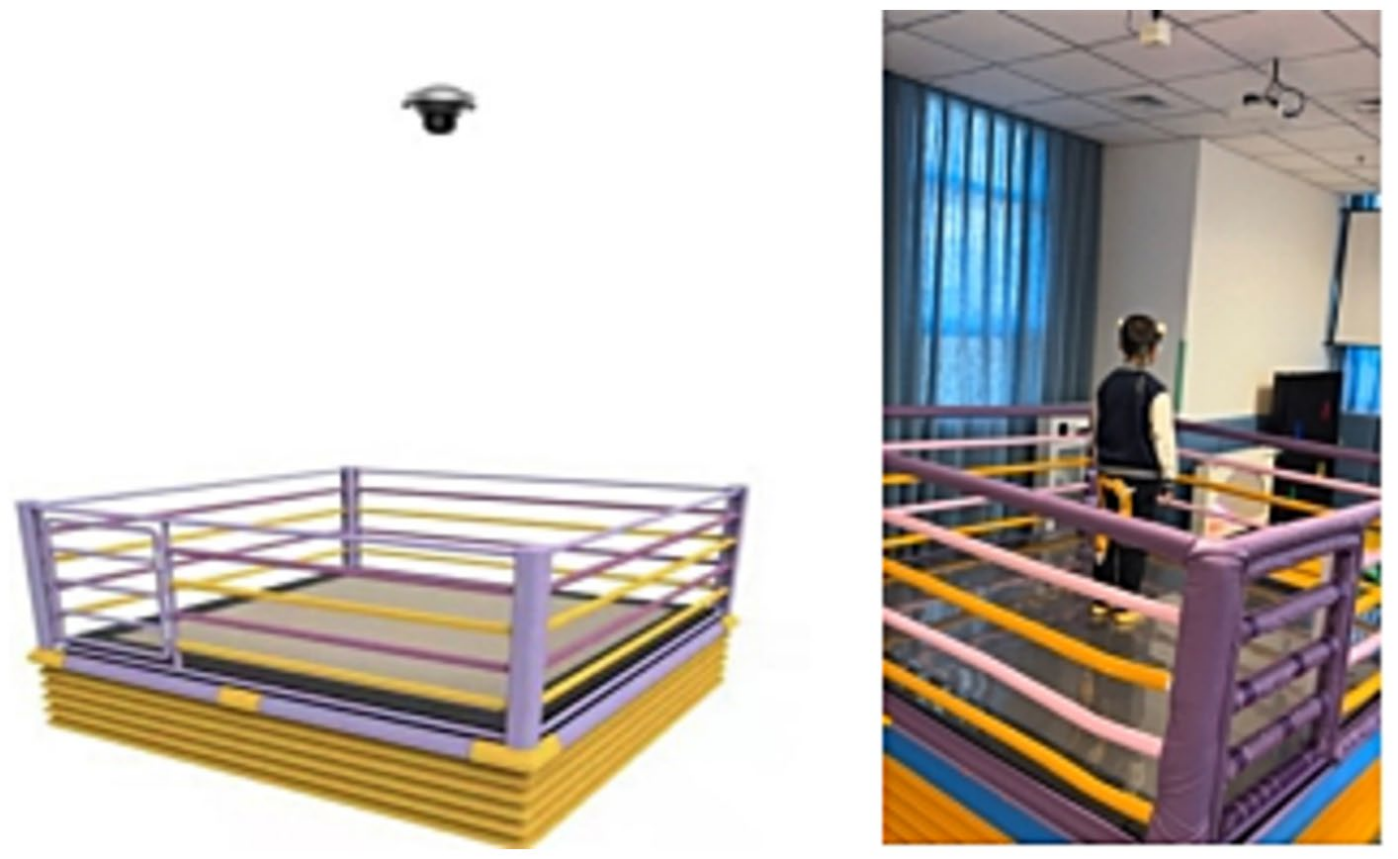
Intelligent multi integrated dynamic training system and human positioning and action recognition analysis system.

**Figure 3:**
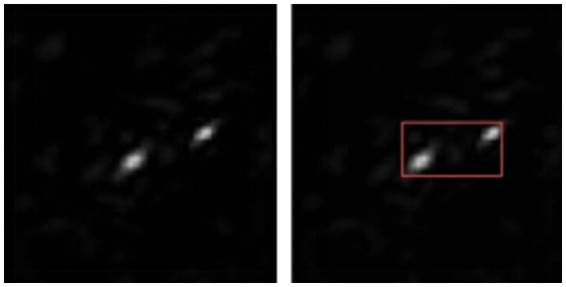
Schematic diagram of the complementary localization algorithm based on MOSSE and infrared recognition.

The balance platform equipment used in the experiment was the Intelligent Multi Integrated Dynamic Training System (Model: GA2480; Haolan Tongxu Technology Co., Ltd.). In speed mode, the platform tilted at a controlled fixed oscillation frequency of 0.1 Hz and amplitude of 5 degrees. The motion capture equipment was the Human Positioning and Action Recognition Analysis System (Model: H-PARA; Haolan Tongxu Technology Co., Ltd.), with a prototype date of October 20, 2023. The three-dimensional camera was positioned directly above the balance platform (Figure 2). The system used head-mounted sensors for identification and positioning and incorporated a MOSSE filtering and infrared recognition algorithm for tracking fusion (Figure 3).

### 2.4 Testing protocol

Testing was conducted in a designated sensory integration therapy room for children with ASD, characterized by quiet conditions, controlled low lighting, and appropriate temperature. Following best practices for sensory-friendly pediatric settings (19), the room included sensory regulation materials such as therapy balls, small trampolines, and swings. Light intensity was controlled using blackout curtains and soft ambient lighting, and auditory distractions were minimized. Before formal testing, children were given time to acclimate to the treatment room environment.

Before each testing session, the motion capture device underwent calibration procedures, including preparation of the testing area, camera and marker setup, adjustment of camera settings, spatial calibration, software configuration, and system optimization testing. Participants were instructed to look straight ahead with both eyes, allow their hands to hang naturally, and position their feet on designated red marker spots on the balance platform to maintain maximum stability. Because children with ASD may experience attentional difficulties, testing was conducted with close monitoring of behavioral state, gentle verbal prompts and encouragement, positive reinforcement, and breaks as needed.

Three postural conditions were tested: long-sitting position (sitting with legs fully extended forward), eyes-open standing position, and eyes-closed standing position. Each condition was tested three times consecutively, and the mean value was calculated for analysis. A 30-s rest period was provided between trials, and a two to three-minute rest period was allowed after completing all trials in each condition to mitigate fatigue effects (20). The order of conditions was randomized using a computer-generated random number sequence to reduce order effects.

During each trial, the balance platform was set to speed mode with the predetermined parameters. The child maintained the selected position on the platform for 1 min. Following three consecutive trials and the rest interval, testing proceeded to the next condition. The identical experimental protocol was repeated 7 days later.

### 2.5 Outcome measures

Image processing techniques were used to determine the 95% confidence ellipse of the head-marker trajectory, yielding three primary outcome measures. These variables quantified head-sway behavior during the balance task and should be interpreted as marker-derived sway indices rather than direct whole-body center-of-mass measurements.

The maximum sway amplitude on the X-axis (Dx) represents the maximum displacement range of the head-marker reference point in the left–right (mediolateral) direction. Larger values indicate greater lateral head-sway during the task.

The maximum sway amplitude on the Y-axis (Dy) represents the maximum displacement range of the head-marker reference point in the forward-backward (anteroposterior) direction. Larger values indicate greater sagittal head-sway during the task.

The trajectory area (Area) represents the ellipse area encompassed by the head-marker trajectory within the specified measurement period, reflecting the overall magnitude of head-sway.

### 2.6 Statistical analysis

#### 2.6.1 Test–retest reliability analysis

Data analysis was conducted using SPSS version 26.0 software. Test–retest reliability was quantified using intraclass correlation coefficients (ICCs) with a two-way mixed-effects model and absolute agreement definition. ICC interpretation followed established guidelines (16): values less than 0.50 indicate poor reliability, values from 0.50 to 0.75 indicate moderate reliability, values from 0.75 to 0.90 indicate good reliability, and values greater than 0.90 indicate excellent reliability. Because the sample size was modest, ICC point estimates were interpreted together with their 95% confidence intervals.

The standard error of measurement (SEM) was calculated to quantify absolute reliability, with higher SEM values indicating greater measurement error (21). Bland–Altman plots were constructed to visualize agreement between the two testing sessions by plotting the difference between measurements against their mean. The mean bias and 95% limits of agreement (95% LOA) were examined, and wider LOA or points outside the LOA were interpreted as evidence of reduced agreement for the corresponding condition or variable.

#### 2.6.2 Exploratory group comparisons and baseline analyses

Data normality was assessed using the Shapiro–Wilk test. For variables demonstrating non-normal distributions, non-parametric tests were employed. The Kruskal-Wallis test was used to analyze differences in balance indicators across the three body positions (long-sitting, eyes-open standing, and eyes-closed standing), with Bonferroni correction applied to post-hoc pairwise comparisons to control for multiple testing.

Unadjusted between-group comparisons of balance indicators for children with ASD versus TD children were conducted using independent samples t-tests for variables meeting normality assumptions and Mann–Whitney U tests for non-normally distributed variables. This combination of parametric and non-parametric tests was selected to align statistical tests with distributional assumptions; however, because each group included only 11 participants, all between-group *p* values and effect sizes were interpreted as exploratory. Bonferroni-corrected significance thresholds were applied for the three balance parameters. Effect sizes were calculated using Cohen’s *d* for normally distributed variables and rank-biserial correlation for non-parametric tests.

Baseline characteristics of the ASD and TD groups, including age, height, weight, BMI, and sex distribution, were compared using independent samples *t*-tests for continuous variables and chi-square tests for categorical variables. Given the small sample size and substantial baseline differences, covariate-adjusted analyses were not performed; the potential confounding effects of age and sex were addressed in the interpretation of the exploratory group comparisons.

## 3 Results

### 3.1 Basic information

This study recruited 22 children, comprising 11 children with ASD and 11 TD children. The overall sample characteristics were: mean age 6.3 ± 2.2 years, mean height 120.6 ± 14.0 cm, and mean weight 23.95 ± 6.7 kg. Detailed demographic information for each group is presented in Tables 1–3. Statistical comparison of baseline characteristics revealed significant differences between the ASD and TD groups. The ASD group was significantly younger than the TD group (mean age: 4.94 ± 1.43 years vs. 7.67 ± 1.92 years; *t* = 3.62, *p* = 0.002). The groups differed significantly in height (*t* = 2.76, *p* = 0.012), but did not differ significantly in weight (*t* = 2.08, *p* = 0.051) or BMI (*t* = 0.70, *p* = 0.49). Regarding sex distribution, no statistically significant difference was observed between groups (χ^2^ = 3.27, *p* = 0.07), although the ASD group comprised a higher proportion of males than the TD group. These baseline imbalances were considered important potential confounders for the exploratory between-group analyses.

### 3.2 Test–retest reliability analysis

#### 3.2.1 Reliability analysis of balance test in children with ASD assessed by motion capture technology

**Figure 4:**
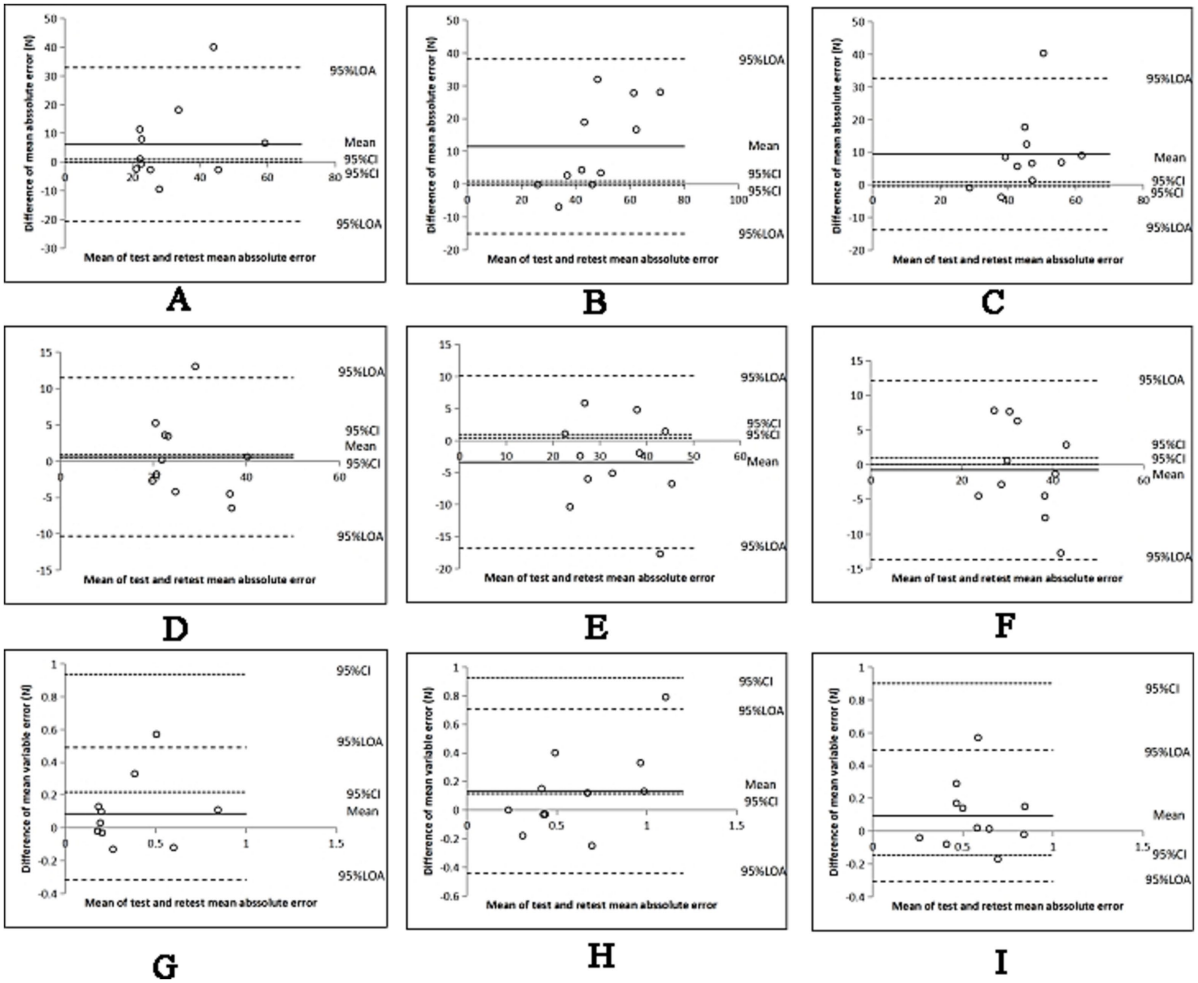
Bland–Altman plots for assessing reliability of the balance test in children with ASD using motion capture technology. **(A)** Dx in long-sitting position; **(B)** Dx in eyes-open standing position; **(C)** Dx in eyes-closed standing position; **(D)** Dy in long-sitting position; **(E)** Dy in eyes-open standing position; **(F)** Dy in eyes-closed standing position; **(G)** Area in long-sitting position; **(H)** Area in eyes-open standing position; **(I)** Area in eyes-closed standing position.

The test–retest reliability results for balance assessment in children with ASD using motion capture technology are presented in Table 4. For Dx across the three positions, ICC values ranged from 0.45 to 0.69, indicating poor to moderate test–retest reliability, with SEM values of 7.64–8.83. The 95% CIs for Dx were wide and included negative lower bounds in all three conditions, indicating unstable estimates and considerable measurement error. For Dy across the three positions, ICC values ranged from 0.77 to 0.87, demonstrating good test–retest reliability, with SEM values ranging from 1.98 to 3.19. The highest reliability was observed for Dy in the long-sitting position (ICC = 0.87). For Area across the three positions, ICC values ranged from 0.65 to 0.77, indicating moderate to good test–retest reliability, with SEM values of 0.10–0.15. SEM values in the long-sitting position were lower than those in the eyes-open and eyes-closed standing positions, suggesting superior measurement precision in the seated posture for this population. Bland–Altman plot analysis for children with ASD showed the Dx and Dy patterns across all three postural conditions (Figures 4A–F, respectively). Points outside the 95% LOA were observed for Dx in the long-sitting and eyes-closed standing conditions (Figures 4A,C), for Dy in the long-sitting and eyes-open standing conditions (Figures 4D,E), and for Area across all three conditions (Figures 4G–I). These outliers and the relatively wide LOA, particularly for Dx, indicate that agreement was not uniform across variables or postural conditions.

**Table 4:** Test–retest reliability results of using motion capture technology to assess the balance of children with ASD.

Position	First test	Second test	Difference ± SD	ICC (95% CI)	SEM	95% LOA
Dx (cm)						
Long sitting position	28.5 ± 9.9	34.6 ± 16.3	−6.1 ± 13.7	0.69(−0.03–0.91)	7.64	−32.9-20.7
Eye open standing position	41.6 ± 6.1	53.1 ± 12.2	−11.5 ± 13.6	0.64(−0.16–0.9)	8.16	−38.1-15.1
Closed eye standing position	40.9 ± 8.4	50.3 ± 18.9	−9.5 ± 11.9	0.45(−0.39–0.83)	8.83	−32.7-13.8
Dy (cm)						
Long sitting position	26.6 ± 0.3	27.2 ± 9.7	−0.6 ± 5.6	0.87(0.52–0.97)	1.98	−11.5-10.3
Eye open standing position	35.1 ± 0.2	31.7 ± 12.4	3.4 ± 6.9	0.82(0.38–0.95)	2.91	−10.1-16.9
Closed eye standing position	34.4 ± 0.4	33.6 ± 8.7	0.8 ± 6.6	0.77(0.09–0.94)	3.19	−12.1-13.7
Area (m^2^)						
Long sitting position	0.3 ± 0.2	0.4 ± 7.4	−0.1 ± 0.2	0.77(0.22–0.94)	0.1	−0.5-0.3
Eye open standing position	0.6 ± 0.2	0.7 ± 8.6	−0.1 ± 0.3	0.74(0.11–0.93)	0.15	−0.7-0.4
Closed eye standing position	0.5 ± 0.2	0.6 ± 8.7	−0.1 ± 0.2	0.65(−0.15–0.9)	0.12	−0.5-0.3

ICC, Intraclass Correlation Coefficient; 95% CI, 95% confidence interval; SEM, Standard Error of Measurement; 95% LOA, 95% limits of agreement. Difference is calculated as first test minus second test. The second test occurred seven days after the first test.

#### 3.2.2 Reliability analysis of balance test in TD children assessed by motion capture technology

**Figure 5:**
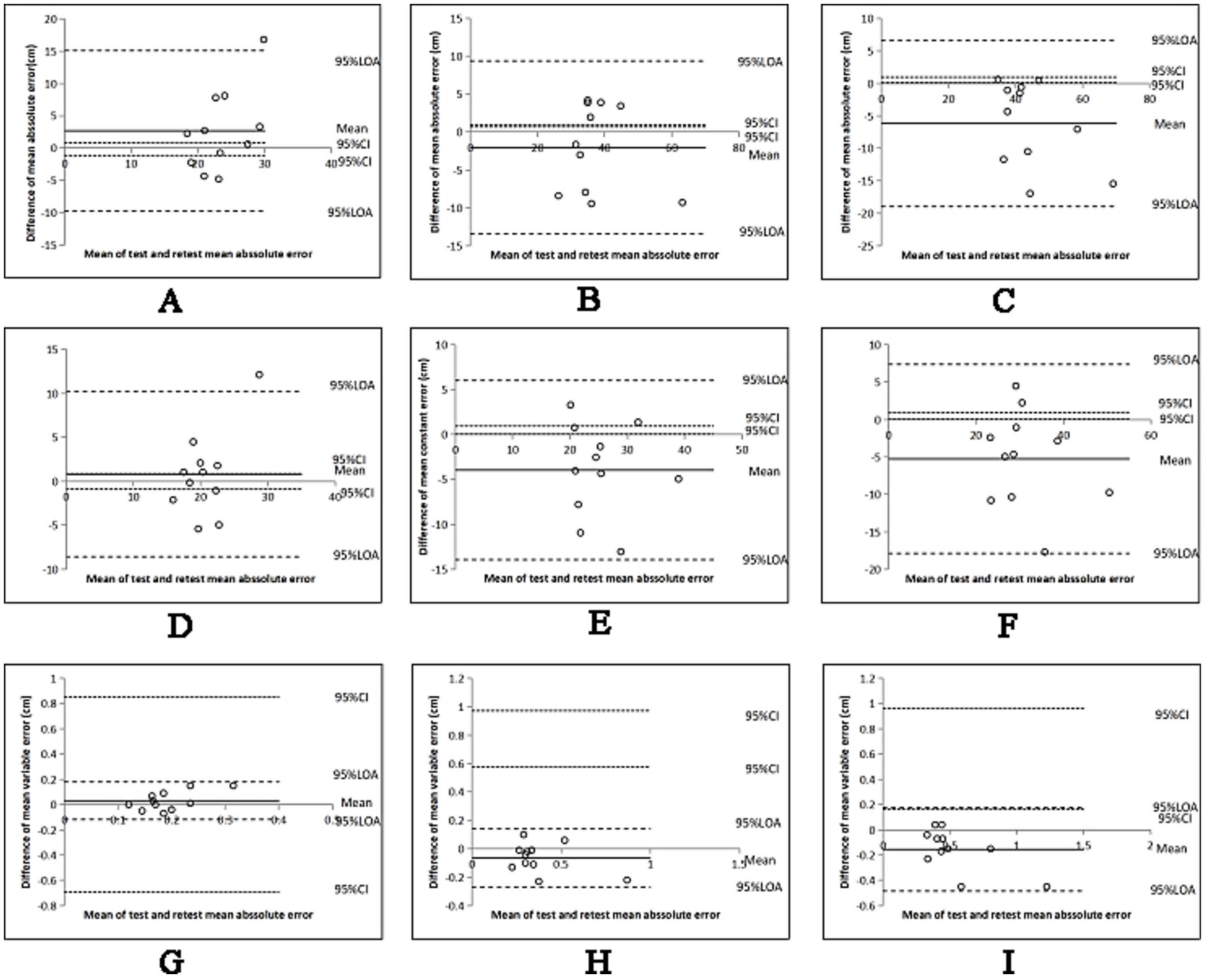
Bland–Altman plots for assessing reliability of the balance test in TD children using motion capture technology. **(A)** Dx in long-sitting position; **(B)** Dx in eyes-open standing position; **(C)** Dx in eyes-closed standing position; **(D)** Dy in long-sitting position; **(E)** Dy in eyes-open standing position; **(F)** Dy in eyes-closed standing position; **(G)** Area in long-sitting position; **(H)** Area in eyes-open standing position; **(I)** Area in eyes-closed standing position.

The test–retest reliability results for balance assessment in TD children using motion capture technology are shown in Table 5. For Dx across the three positions, ICC values ranged from 0.31 to 0.91, indicating poor to excellent test–retest reliability, with SEM values ranging from 1.00 to 5.25. For Dy across the three positions, ICC values ranged from 0.53 to 0.76, demonstrating moderate to good test–retest reliability, with SEM values of 2.62–3.28. For Area across the three positions, ICC values ranged from 0.48 to 0.89, indicating poor to good reliability, with SEM values of 0.03–0.07. In contrast to the ASD group, SEM values in the eyes-open standing position were lower than those in the long-sitting and eyes-closed standing positions for TD children, suggesting that the eyes-open standing position produced the most precise measurements in this group. Bland–Altman analysis revealed differences outside the 95% LOA for Dx and Dy in the long-sitting condition (Figures 5A,D), whereas no such outliers were observed for Dx or Dy in either standing position (Figures 5B,C,E,F). For Area measurements, all individual differences across the three postural conditions fell within the 95% LOA limits (Figures 5G–I).

**Table 5:** Test–retest reliability results of using motion capture technology to assess the balance of TD children.

Position	First test	Second test	Difference ± SD	ICC (95% CI)	SEM	95% LOA
Dx (cm)						
Long sitting position	22.2 ± 6.6	24.9 ± 6.2	−2.7 ± 6.3	0.31(−1.15–0.81)	5.25	−15.1-9.7
Eye open standing position	38.6 ± 7.7	36.5 ± 3.5	2.0 ± 5.8	0.91(0.67–0.97)	1.79	−9.4-13.5
Closed eye standing position	47.8 ± 9.2	41.6 ± 9.7	6.2 ± 6.5	0.84(0.13–0.96)	2.65	−6.6-19.0
Dy (cm)						
Long sitting position	20.2 ± 0.1	21.0 ± 10.4	−0.8 ± 4.8	0.53(−0.87–0.88)	3.28	−10.2-8.6
Eye open standing position	27.4 ± 0.0	23.5 ± 9.3	4.0 ± 5.1	0.73(0.03–0.93)	2.62	−6.0-13.9
Closed eye standing position	33.9 ± 0.2	28.6 ± 12.3	5.3 ± 6.4	0.76(0.06–0.94)	3.16	−7.4-17.9
Area (m^2^)						
Long sitting position	0.2 ± 0.2	0.2 ± 5.2	−0.0 ± 0.1	0.48(−0.69–0.85)	0.05	−0.2 − 0.1
Eye open standing position	0.4 ± 0.2	0.3 ± 2.9	0.1 ± 0.1	0.89(0.58–0.97)	0.03	-0.1-0.3
Closed eye standing position	0.6 ± 0.3	0.5 ± 6.1	0.2 ± 0.2	0.84(0.16–0.96)	0.07	−0.2-0.5

ICC, Intraclass Correlation Coefficient; 95% CI, 95% confidence interval; SEM, Standard Error of Measurement; 95% LOA, 95% limits of agreement. Difference is calculated as first test minus second test. The second test occurred 7 days after the first test.

### 3.3 Exploratory group comparisons

#### 3.3.1 Comparison of balance indicators among postural conditions

Normality testing using the Shapiro–Wilk test revealed that the balance indicators Dx, Dy, and Area demonstrated non-normal distributions across the three positions. Consequently, non-parametric testing using the Kruskal-Wallis test was employed to analyze differences in the total sample (*N* = 22, comprising both ASD and TD groups). Analysis revealed statistically significant differences (*p* < 0.01) across the three balance indicators of Dx, Dy, and Area among the three different positions (Table 6). Bonferroni-corrected post-hoc pairwise comparisons revealed significant differences between the long-sitting position and both the eyes-open and eyes-closed standing positions for all three balance parameters (all adjusted *p* < 0.01). No significant differences were observed between the eyes-open and eyes-closed standing positions after correction for multiple comparisons (all adjusted *p* > 0.05). Based on the measured balance indicator values, the long-sitting position demonstrated greater postural stability than both standing positions, consistent with the biomechanical advantage of a larger base of support in the seated posture.

**Table 6:** Analysis of differences in balance indicators among three positions.

Balance indicator	Position median (P25, P75)			Kruskal-Wallis Test statistic *H*-value	*p*
	Long sitting position (*n* = 22)	Eye open standing position (*n* = 22)	Closed eye standing position (*n* = 22)		
Dx (cm)	25.28 (21.94, 32.74)	39.53 (31.05, 54.11)	44.55 (36.08, 54.11)	19.612	<0.001**
Dy (cm)	21.83 (19.46, 27.08)	24.53 (21.59, 34.65)	31.20 (25.73, 35.55)	9.650	0.008**
Envelope area (m^2^)	0.21 (0.17, 0.33)	0.38 (0.26, 0.70)	0.51 (0.37, 0.67)	17.692	<0.001**

*p < 0.05, **p < 0.01. Bonferroni-corrected post-hoc comparisons revealed significant differences between long-sitting and both standing positions, but not between eyes-open and eyes-closed standing positions.

#### 3.3.2 Exploratory comparison of balance indicators between ASD and TD groups

Normality testing indicated that Dy and Area for both the ASD and TD groups exhibited non-normal distributions, necessitating non-parametric testing, while Dx demonstrated approximate normal distribution, permitting independent samples t-test analysis. In unadjusted exploratory comparisons, significant differences (*p* < 0.01) were observed across Dx, Dy, and Area between the ASD and TD groups (Table 7), and these comparisons remained significant after Bonferroni correction for the three balance parameters. Effect size calculations revealed that the effect size for Dx was 0.795, while effect sizes for Dy and Area were 0.310 and 0.272, respectively. These findings indicate that balance-related head-sway indices differed between the groups in this sample. However, because the groups were not matched for age or sex and the study was powered for reliability rather than group discrimination, these results should be regarded as exploratory and should not be interpreted as diagnostic evidence.

**Table 7:** Exploratory unadjusted comparison of balance indicators between children with ASD and TD children.

Group/statistic	Dx (cm)	Dy (cm)	Envelope area (m^2^)
ASD group (*n* = 11)	45.99 ± 17.62	31.05 (23.70, 35.78)	0.55 (0.25, 0.76)
TD group (*n* = 11)	34.34 ± 10.90	22.35 (19.58, 27.75)	0.30 (0.21, 0.40)
*t* (z)	−3.23	−3.412	−2.996
*p*	0.002**	<0.001**	0.003**
Effect size	0.795	0.310	0.272

*p < 0.05, **p < 0.01. Effect sizes were calculated using Cohen’s *d* for Dx and rank-biserial correlation for Dy and Area. These analyses were not powered for definitive between-group inference and were not adjusted for age or sex; therefore, they should not be interpreted as diagnostic evidence.

## 4 Discussion

### 4.1 Summary of principal findings

This study evaluated the test–retest reliability of a portable marker-based motion capture system for assessing head-sway-derived balance indices in children with ASD and TD children. The principal finding was that anteroposterior sway amplitude (Dy) showed good reliability in children with ASD across the three postural conditions, with the highest ICC and lowest SEM observed in the long-sitting position. Dx showed poorer and more variable reliability, and Area showed moderate to good reliability. Reliability patterns differed in TD children, with the eyes-open standing condition showing the lowest SEM for several parameters. Unadjusted between-group comparisons showed larger sway values in the ASD group, but these analyses were exploratory and potentially confounded by age and sex differences.

### 4.2 Interpretation in relation to existing literature

Children with ASD often experience motor and balance difficulties, and structured physical activity interventions, including martial arts and other exercise-based approaches, have been reported to improve motor skills and balance-related outcomes (22, 23). Balance control depends on coordinated integration of visual, vestibular, proprioceptive, musculoskeletal, and sensorimotor systems (24). Reliable measurement of balance is therefore important for clinical assessment and intervention planning, but psychometric properties must be established before a tool can be interpreted confidently (25).

The ICC values for Dy in children with ASD (0.77–0.87) indicate good reliability according to established reliability thresholds (16). These results suggest that the portable system can reproducibly capture anteroposterior head-sway amplitude over a one-week interval in this small ASD sample. In contrast, the reliability of Dx was lower and the confidence intervals were wide, indicating that mediolateral head-sway was more variable across repeated sessions. Prior research has emphasized that sensory integration and attentional demands can influence postural control in ASD (26), and developmental studies have reported differences in postural stability in children with ASD compared with TD peers (27). The present reliability findings are consistent with the view that balance performance in ASD is variable and that not all sway directions or postural conditions yield equally stable measurements.

The long-sitting condition produced the most precise Dy measurement in children with ASD. This may reflect the larger base of support and lower postural challenge of the seated posture, which can reduce behavioral and biomechanical variability during testing. The eyes-open standing condition appeared more precise in TD children, likely because it more closely reflects a familiar functional posture. These interpretations should be considered hypotheses generated from the reliability pattern rather than causal explanations, because the present study was not designed to isolate sensory, behavioral, or biomechanical mechanisms.

### 4.3 Measurement validity and Bland–Altman interpretation

A key methodological issue is the distinction between reliability and validity. The system demonstrated reproducibility for selected head-sway-derived indices, particularly Dy, but this does not establish that the indices are valid measures of whole-body center-of-mass displacement or clinical balance impairment. The head-mounted markers provide a pragmatic reference point for head motion; however, head movement can differ from whole-body center-of-mass movement, especially in children with atypical motor control strategies. Therefore, the current results support reliability of the captured marker trajectory, whereas construct and concurrent validity require further testing against reference methods such as force platforms or full-body motion capture.

The Bland–Altman findings also require cautious interpretation. Although mean differences were generally close to zero for several variables, points outside the 95% LOA were observed in multiple ASD conditions and in long-sitting Dx and Dy among TD children. In addition, several LOA were wide relative to the mean values, particularly for Dx. These patterns indicate that agreement was not uniformly strong across outcomes and that individual-level change scores, especially for Dx, may be difficult to interpret without additional validation and clinically meaningful change thresholds. Bland–Altman plots are useful not only for identifying systematic bias but also for judging whether the observed limits of agreement are acceptable for the intended clinical application (28).

### 4.4 Clinical implications

The findings suggest that portable motion capture may be useful for repeated assessment of anteroposterior head-sway in children with ASD, particularly when a standing task is difficult or anxiety-provoking. Children with ASD may show sedentary behavior patterns and variable engagement during motor assessment (29), making a more stable long-sitting option clinically relevant. Nevertheless, the present system should not yet be used as a diagnostic tool or as a stand-alone measure of balance impairment. Its most defensible current application is as a preliminary quantitative method for monitoring reproducible head-sway indices under standardized conditions while additional validity and responsiveness evidence is accumulated.

### 4.5 Limitations and future directions

Several limitations should be considered. First, the sample size was modest, although it met the *a priori* target for reliability estimation. With only 11 participants per group, ICC confidence intervals and exploratory group-difference estimates were unstable, and the study was not powered for definitive between-group inference. Second, the ASD and TD groups differed substantially in age and sex distribution. Because postural control develops throughout childhood and may be influenced by sex-related motor development, the unadjusted group comparisons cannot be attributed confidently to ASD status. Future studies should use age- and sex-matched samples or adequately powered covariate-adjusted analyses.

Third, the head-mounted marker approach measured head-sway rather than true whole-body center-of-mass movement. Validation against force platforms, pressure platforms, or full-body motion capture is needed to determine the clinical meaning of Dx, Dy, and Area. Fourth, only one motion capture system and one set of platform perturbation parameters were examined, limiting generalizability to other technologies and stimulus conditions. Fifth, reliability was assessed over a single seven-day interval; additional intervals would clarify short- and longer-term temporal stability. Future research should also establish responsiveness to intervention, minimal detectable change, clinically meaningful change thresholds, and predictive validity for functional outcomes such as fall risk, participation, or motor skill development. These steps are necessary before broad clinical implementation can be recommended (30–33).

## 5 Conclusion

This study provides preliminary evidence for the test–retest reliability of a portable marker-based motion capture system for assessing head-sway-derived dynamic balance indices in children with ASD across a seven-day interval. In children with ASD, anteroposterior sway amplitude (Dy) demonstrated good reliability (ICC = 0.77–0.87) with relatively low measurement error (SEM = 1.98–3.19), and the long-sitting position produced the most precise measurements. Dx and Area showed more variable reliability and should be interpreted more cautiously. Exploratory unadjusted comparisons indicated differences in balance-related head-sway indices between ASD and TD groups, but these findings cannot be interpreted as diagnostic or discriminative evidence because of the small sample size and baseline age and sex differences. The portability, ease of operation, and relatively low cost of this system support its further evaluation as a quantitative tool for repeated balance assessment in pediatric ASD settings. Future studies should validate these head-sway indices against force-plate or full-body motion capture standards, recruit larger age- and sex-matched samples, examine responsiveness to intervention, and determine clinically meaningful change thresholds before routine clinical implementation is recommended.

## Data Availability

The original contributions presented in the study are included in the article/supplementary material, further inquiries can be directed to the corresponding authors.
